# 15 years of BMC Biology

**DOI:** 10.1186/s12915-018-0602-8

**Published:** 2018-11-01

**Authors:** Christian Matheou, Graham P. Bell, Penelope Austin, Vitor Sousa, Mirna Kvajo

**Affiliations:** Springer Nature, New York, USA

Launched in November 2003 as the flagship Open Access journal of the BMC Series, *BMC Biology*—which this year celebrates its 15th anniversary—continues to provide a home for outstanding research in all fields of biology. Buoyed by the pioneering spirit of BMC and its dedicated editorial team, *BMC Biology* has shaped itself through innovation in publishing and a commitment to the highest standards of author service.

To celebrate this occasion, we shall be revisiting moments that, over the past 15 years, have helped build *BMC Biology*, and what we feel are our defining features. We are doing so through a series of commissioned pieces, and a *timeline* that marks the history of our journal.

Keen to meet the changing needs of the scientific community, *BMC Biology* has been an eager innovator, adopting policies and practices designed to reduce waste of time and effort in publishing. Re-review opt out, portable peer review, and initiatives geared towards improving reproducibility allowed us to publish impactful and rigorous studies while offering authors a better publication experience. As editors, these policies also remind us that thinking outside the box has the power to make publishing better, and inspire us to think of ways to tackle the next challenges.

A distinguishing mark of *BMC Biology* is our formidable Editorial Board. Over the years, our editorial board members helped to establish the journal, and continue to serve as trusted advisors, sources of innovative ideas, and guides for our inroads into new fields. We get to benefit from their authoritative voices, and on this occasion we are delighted to share them with you through a series of Open Questions that bring the reader to the frontiers of our editorial board members’ respective fields. These comments explore topics in search of answers, such as what we still need to understand about the biology of CRISPR, how many genes we actually have, what are respiratory chain supercomplexes good for, and others. We hope these Open Questions (and others, that you can read *here*) will spark your curiosity and inspire your own research.

Authors’ lives and career paths, their inspirations, successes, and failures, underpin the research that we and other journals publish, yet these largely stay behind the scenes—and for every paper that gets published, there are many untold stories. To provide a voice to these stories, and draw on the wisdom of our past authors, we interviewed a number of scientists who have published on the pages of our journal. Their profiles provide a sketch of what is it like to be a scientist today, the questions that inspire their research and that are still open in their fields, their lab life, and advice they would have given themselves, and would now like to offer to others. We hope that our readers—from trainees to senior scientists—see themselves reflected in these portraits and find them motivating, and useful.

Peer review is the cornerstone of publishing, and we could not rightly celebrate the journal’s anniversary without acknowledging the invaluable contribution and efforts of our reviewers throughout the years. Included in this collection are interviews with a selection of past reviewers (who have in some cases also published with us as authors) from various fields, who offer their thoughts on the current state of peer review. Especially with the increased numbers, length, and complexity of submissions, we would like to extend our thanks to all of our conscientious and dedicated reviewers.

In 2003 *BMC Biology* was launched as a vanguard of a big new idea in publishing—the Open Access movement. Fifteen years later, Open Access is not so radical. But, in the intervening years we have seen a renewed focus on open science and reproducibility, the arrival of preprint servers, and a growing awareness that journals need be able to distinguish themselves in the way that they serve authors and science as a whole. What’s in store for the next 15 years? It is hard to tell, but we do know that we will be here to bring even more outstanding science to our readers, while continuing our commitment to bright new ideas.

